# Genome-Wide Detection of Fitness Genes in Uropathogenic *Escherichia coli* during Systemic Infection

**DOI:** 10.1371/journal.ppat.1003788

**Published:** 2013-12-05

**Authors:** Sargurunathan Subashchandrabose, Sara N. Smith, Rachel R. Spurbeck, Monica M. Kole, Harry L. T. Mobley

**Affiliations:** Department of Microbiology and Immunology, University of Michigan Medical School, Ann Arbor, Michigan, United States of America; Tufts University School of Medicine, United States of America

## Abstract

Uropathogenic *Escherichia coli* (UPEC) is a leading etiological agent of bacteremia in humans. Virulence mechanisms of UPEC in the context of urinary tract infections have been subjected to extensive research. However, understanding of the fitness mechanisms used by UPEC during bacteremia and systemic infection is limited. A forward genetic screen was utilized to detect transposon insertion mutants with fitness defects during colonization of mouse spleens. An inoculum comprised of 360,000 transposon mutants in the UPEC strain CFT073, cultured from the blood of a patient with pyelonephritis, was used to inoculate mice intravenously. Transposon insertion sites in the inoculum (input) and bacteria colonizing the spleen (output) were identified using high-throughput sequencing of transposon-chromosome junctions. Using frequencies of representation of each insertion mutant in the input and output samples, 242 candidate fitness genes were identified. Co-infection experiments with each of 11 defined mutants and the wild-type strain demonstrated that 82% (9 of 11) of the tested candidate fitness genes were required for optimal fitness in a mouse model of systemic infection. Genes involved in biosynthesis of poly-N-acetyl glucosamine (*pgaABCD*), major and minor pilin of a type IV pilus (*c2394* and *c2395*), oligopeptide uptake periplasmic-binding protein (*oppA*), sensitive to antimicrobial peptides (*sapABCDF*), putative outer membrane receptor (*yddB*), zinc metallopeptidase (*pqqL*), a shikimate pathway gene (*c1220*) and autotransporter serine proteases (*pic* and *vat*) were further characterized. Here, we report the first genome-wide identification of genes that contribute to fitness in UPEC during systemic infection in a mammalian host. These fitness factors may represent targets for developing novel therapeutics against UPEC.

## Author Summary

Uropathogenic *E. coli* is a major cause of bacterial bloodstream infections in humans. Dissemination of *E. coli* into the bloodstream during urinary tract infections may lead to potentially fatal complications. This pathogen is becoming increasingly resistant to currently used antibiotics. To develop additional tools to treat such infections, a thorough understanding of the mechanism of pathogenesis is required. Here, we report major progress towards that goal by identifying bacterial genes that are critical for the ability of this pathogen to cause bloodstream infections using a mouse model of infection. This study sheds light on the conditions encountered by *E. coli* during systemic infection. Further research on the genes identified in this study may reveal bacterial targets that can be used to develop novel therapeutics against bloodstream infections caused by *E. coli*.

## Introduction

Uropathogenic *Escherichia coli* (UPEC), one of the most common bacterial pathogens infecting humans, is the primary etiological agent of urinary tract infections (UTI) in otherwise healthy individuals [1]. UPEC is a subset of extraintestinal pathogenic *E. coli* (ExPEC), which causes a broad spectrum of conditions including colibacillosis in poultry, and UTIs, bacteremia, and neonatal meningitis in humans [2]. A subset of patients with UTI develops pyelonephritis and is at risk for developing bacteremia that may result in life threatening sepsis. UTI is the source of *E. coli* in >70% of both young and elderly patients with bloodstream infections [3], [4]. *E. coli* strains isolated from the bloodstream are becoming increasingly resistant to trimethoprim/sulfamethoxazole and ciprofloxacin, two first line antibiotics used to treat bacterial UTIs [5]. Despite the prevalence of these infections and potential difficulties in treatment, little is known about the fitness and virulence mechanisms employed by *E. coli* to establish a systemic infection.

The marriage between transposon mutagenesis and high-throughput (HT) sequencing has resulted in the emergence of powerful techniques that can be harnessed for global functional genomic studies [6]. Here, we utilize an adaptation of transposon directed insertion-site sequencing (TraDIS) [7] to identify genes required for optimal fitness of UPEC during colonization and survival in a murine model of bacteremia. Recently, such approaches were used to determine virulence and fitness factors in *Yersinia pseudotuberculosis* [8] and *Salmonella enterica* serovar Typhimurium [9] utilizing animal models of infection and colonization.

Genes that encode microbial proteins and organelles that specifically aid in pathogenesis are known as virulence genes. Bacterial pathogens are adept at co-opting genes that are otherwise used in non-pathogenesis related roles for gaining fitness advantage during infection. In this context, fitness refers to enhanced survival and growth within a given niche. Genes that promote colonization and survival of UPEC within murine hosts are referred to as fitness factors in this manuscript. A subset of the fitness factors reported here, represent virulence factors that meet the criteria defined by molecular Koch's postulates [10]. This report, to our knowledge, represents the first global functional genomic screen aimed at identification of *in vivo* fitness factors in a pathogenic *E. coli* strain involving a targeted-sequencing approach.

In this study, a murine model of invasive UPEC infection, previously developed in our laboratory [11], was used in conjunction with transposon mutagenesis to identify bacterial fitness mechanisms involved in establishing systemic infection. Mice were inoculated intravenously with an inoculum derived from a saturating transposon mutant library of a clinical bacteremia isolate, *E. coli* CFT073. Transposon mutants that colonized and survived in mouse spleens (output) were isolated. Transposon insertion sites in the input and output samples were mapped to the genome of the UPEC strain CFT073. 242 candidate fitness genes that are required for optimal survival in the spleen were identified in the primary screen. Genetically defined mutants were constructed and tested for *in vivo* and *in vitro* fitness phenotypes using assays relevant to the infection biology of UPEC. A subset of these fitness factors are also involved in the development of UTI in a mouse model and suggests the existence of shared fitness mechanisms used at these disparate body sites. In summary, we present a comprehensive study of fitness factors that augment the survival of UPEC during systemic disseminated infection in a mammalian host.

## Results

### Generation of a transposon mutant library

*E. coli* CFT073, isolated from the urine and blood of a patient hospitalized with pyelonephritis and bacteremia [12], was used to construct a high-density transposon mutant library. An estimated 48,174 transposon mutants are required to obtain a 99.99% saturation of the CFT073 genome [13], which is 5.2 Mbp in length [14]. A genome-supersaturating Tn*5* transposon mutant library, containing 360,000 kanamycin-resistant transformants, was generated for this study. The library was passaged three times in lysogeny broth (LB) to enrich for mutants that did not exhibit a fitness defect *in vitro*. This enriched mutant pool was used as the inoculum for infection experiments.

### Primary screen of the transposon mutant library in mice

**Figure 1:**
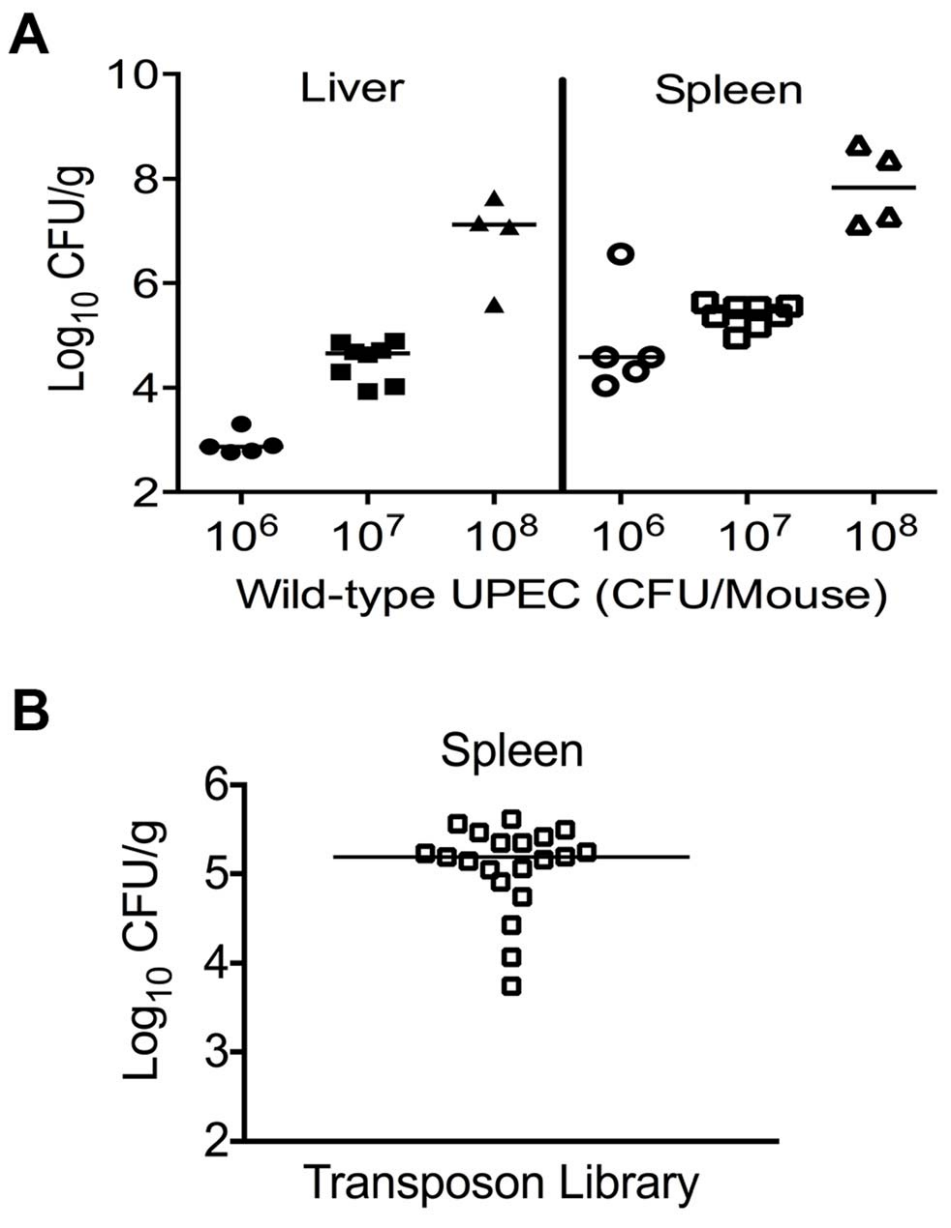
Colonization of uropathogenic *E. coli* during systemic disseminated infection in mice. (A) Bacterial load in the spleens and livers of mice at 24 hpi. CBA/J mice were inoculated with indicated doses of the wild-type *E. coli* strain CFT073 via the tail vein. Each data point indicates CFU count obtained from an individual mouse. (B) Twenty mice were inoculated with Tn*5* transposon mutant pool and the bacterial load in spleens is depicted. These colonies were harvested for genomic DNA extraction and used for determination of transposon insertion sites by targeted sequencing.

A murine model of systemic disseminated UPEC infection [11] was used to determine the highest dose of wild-type CFT073 that consistently resulted in non-lethal infection. Three doses (10^6^, 10^7^ and 10^8^ CFU/mouse) were compared in the CBA/J mouse model of systemic disseminated infection. Mice were inoculated via tail vein, and livers and spleens were collected 24 h post inoculation (hpi). An inoculum of 10^7^ CFU resulted in consistent colonization without causing distress in the inoculated animals (Fig. 1A). Inoculation with 10^6^ CFU led to poor colonization, whereas a dose of 10^8^ CFU resulted in 20% mortality (Fig. 1A). Twenty mice were inoculated with 10^7^ CFU of transposon-insertion mutants (input) and euthanized 24 hpi (Fig. 1B). As a major reticuloendothelial organ, the spleen is a critical site of active bacterial killing during systemic infection [15]. Therefore, a bacterium that successfully survives in the spleen should contain the full complement of fitness factors that are critical for survival in that niche, including the ability to overcome host defenses activated during systemic bacterial infection. Bacteria that grew from splenic homogenates were harvested (output) and used to isolate genomic DNA for Illumina sequencing.

### Identification of transposon insertion sites

Transposon insertion sites in the inoculum (input) and bacteria colonizing the spleens (output) were determined using transposon directed insertion-site sequencing (TraDIS), a HT sequencing-based approach [7]. Genomic DNA from the input and output samples were used to generate TruSeq sequencing libraries (Illumina). Libraries were amplified using a transposon-specific forward primer and a custom adapter-specific reverse primer (Table S1). Resulting amplicons were used for cluster generation and each library was sequenced with a Tn-specific primer (Table S1) in an IIlumina HiSeq 2000 sequencer. Fifty nucleotide single-end reads in FASTQ format were aligned to the *E. coli* CFT073 genome [14] using the short read aligner, BOWTIE [16]. The number of reads processed was 75,935,499 and 87,030,926 for the input and output samples, respectively. 76.7% (58,209,557) of the reads from input and 77.9% (67,810,765) of the reads from output were aligned unambiguously to the CFT073 genome. TFAST [17] was used to determine the exact genomic location of the Tn-insertion and the frequency of reads that map to a given insertion site.

### Determination of candidate fitness genes

**Figure 2:**
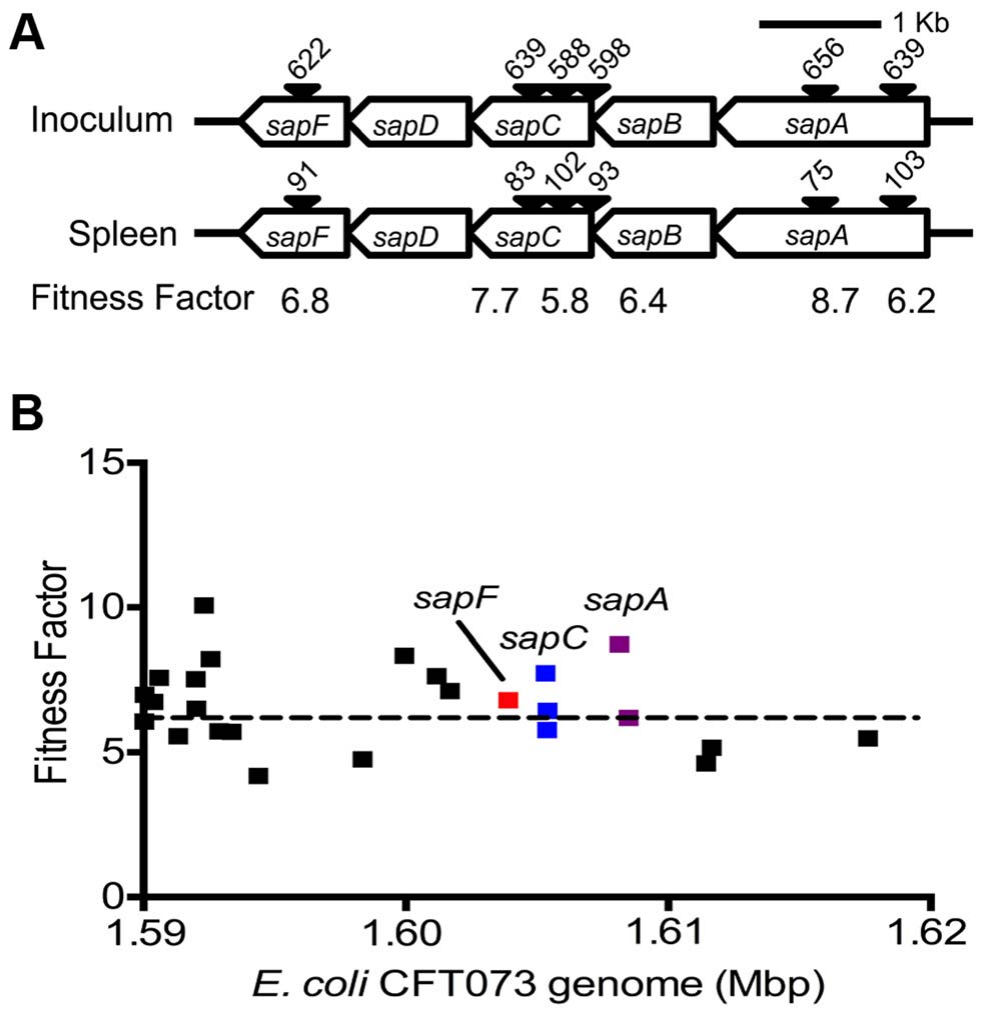
Determination of *in vivo* fitness factors. (A) Transposon insertion sites (filled arrow heads) that map to a specific location in the *sap* gene cluster in the *E. coli* CFT073 genome are depicted along with the number of normalized reads. Fitness factors were calculated as the ratio of number of reads that map to a specific insertion site in the inoculum (input) to the number of reads that map to the same insertion site in the splenic cultures (output). (B) To better visualize the coverage of transposon mutagenesis, a 30 Kbp window with 25 mapped Tn-insertion sites that exhibit a range of fitness factors is illustrated. Dotted line indicates the threshold (mean+2 standard deviations) used to delineate fitness genes. Fitness factors corresponding to the insertion sites within *sapF* (red), *sapC* (blue) and *sapA* (purple) genes are denoted.

Reads for each transposon insertion site were normalized to the total number of reads obtained from that sample and a fitness factor was calculated for each Tn-insertion mutant as the ratio of normalized frequency of reads in the input to that of the output (Fig. 2A, Table 1 and Table S2). Therefore, a fitness factor ≥1 indicates that a given mutant is underrepresented in the output pool. For example, the *s*ensitive to *a*ntimicrobial *p*eptide (*sap*) gene cluster in *E. coli* CFT073 is depicted along with the frequency of representation of transposon-insertion mutants in the input and the output pools (Fig. 2A). Transformants containing an insertion in the *sap* genes are less well represented in the output pool compared to the input pool. A total of 6732 unique Tn*5* insertion sites were mapped in the CFT073 genome (Table S2) with a mean fitness factor of 3.27±1.57. The insertion sites were distributed throughout the length of the CFT073 genome. At least a single transposon insertion site was observed in 3020 genes and an additional 843 intergenic regions, which could exert polar effects of downstream genes. A relatively short region in the genome (30 Kbp in length) reveals the presence of transposon insertion mutants with a broad range of fitness factors (Fig. 2B). The median distance between independent insertion sites was 561 bp. A total of 372 transposon insertion mutants, resulting in inactivation of 242 genes, exhibited fitness factors >6.41 (mean+2 standard deviations) and were considered as candidate fitness genes. 50 transposon mutants with the highest *in vivo* fitness defect phenotype are listed in Table 1. Seven (1.9%) of 372 candidate transposon mutants were previously designated as essential genes in a laboratory strain of *E. coli* K-12 [18]. These genes encode NrdB, an aerobic ribonucleotide reductase; MviN, a peptidoglycan lipid II flippase; LolD, lipoprotein releasing system ATP-binding protein; MinD, septum site-determining protein; GapA, glyceraldehyde-3-phosphate dehydrogenase; FabG, 3-ketoacyl-acyl carrier protein reductase; and MsbA, lipid flippase.

**Table 1:** Transposon mutants with reduced *in vivo* fitness.

Gene	CDS	Annotation	Fitness Factor
	*c1220*	3-deoxy-D-*arabino*-heptulosonic acid-7-phosphate synthase	12.92
*nrdB*	*c2777*	Ribonucleotide reductase	11.68
*yeiA*	*c2680*	Dihydropyrimidine dehydrogenase	11.59
		Between hypothetical proteins encoded by *c1270* and *c1271*	11.42
*ybcM*	*c2349*	Putative transcriptional regulator	11.32
	*c2492*	Putative carbohydrate/ribose kinase	10.95
	*c1891*	Hypothetical protein	10.89
	*c2392*	Putative p4-like integrase	10.83
*yegW*	*c2628*	GntR-type regulator	10.66
*ydhK*	*c2037*	Predicted membrane protein (putative fusaric acid resistance)	10.48
*ydcL*	*c1856*	Lipoprotein precursor	10.16
*rnb*	*c1757*	Exoribonuclease II (mRNA degradation)	10.06
*cfa*	*c2055*	Cyclopropane fatty acyl phospholipid synthase	9.98
*ycgF*	*c1606*	EAL domain protein	9.69
*fadD*	*c2209*	Long chain fatty acid-CoA ligase	9.64
	*c2608*	Hypothetical protein (predicted membrane protein)	9.56
*cobS*	*c2478*	Cobalamine synthase	9.50
	*c2511*	Transposase insertion sequence protein	9.49
*ydcP*	*c1859*	Protease	9.43
*pgaB*	*c1162*	Periplasmic deacetylase	9.41
*yegR*	*c2613*	Hypothetical protein	9.38
*ydcP*	*c1859*	Protease	9.27
*ydhK*	*c2037*	Predicted membrane protein (putative fusaric acid resistance)	9.26
*ptrB*	*c2256*	Protease II	9.24
*yddB*	*c1924*	Outer membrane protein (putative tonB dependent receptor)	9.20
*fumA*	*c2004*	Fumarate hydratase class I, aerobic	9.17
*iscR*	*c3057*	Transcriptional regulator of the *iscRSUA* operon	9.16
*rstB*	*c2001*	Sensor protein, RstAB two-component system	9.14
*yddB*	*c1924*	Outer membrane protein (putative tonB dependent receptor)	9.14
*trxB*	*c1025*	Thioredoxin reductase	9.09
	*c2512*	Transposase insertion sequence protein	9.09
*ynbD*	*c1837*	Inner membrane phosphatase	9.06
*yegN*	*c2601*	Multidrug efflux system subunit MdtB	9.03
*ychK*	*c1698*	Ortholog of RssA, a putative phospholipases	8.99
*ycjU*	*c1789*	Putative beta-phosphoglucomutase	8.99
*wcaL*	*c2569*	Colanic acid biosynthesis glycosyl transferase	8.89
	*c2511*	Transposase insertion sequence protein	8.88
*cobW*	*c1267*	Hypothetical protein (putative cobalamin synthesis protein)	8.85
*guaB*	*c3027*	Inosine 5′-monophosphate dehydrogenase	8.77
*ycjU*	*c1789*	Putative beta-phosphoglucomutase	8.75
	*c2511*	Transposase insertion sequence protein	8.75
*sapA*	*c1771*	Peptide transport periplasmic protein	8.73
		Between tRNAs, ValX and ValY	8.73
*ydgI*	*c1997*	Putative arginine/ornithine antiporter	8.72
*ynfC*	*c1975*	Hypothetical protein	8.62
	*c2393*	Hypothetical protein	8.62
*yeeO*	*c2448*	Putative efflux protein of MatE family	8.56
*ychK*	*c1698*	Ortholog of RssA, a putative phospholipases	8.54
*narX*	*c1682*	Nitrate/nitrite sensor protein	8.49
	*c1689*	Putative 3-oxoacyl (acyl carrier protein) synthase III	8.47

### Nucleotide metabolism

Genes involved in nucleotide metabolism were previously described to play a critical role in growth of a non-pathogenic strain of *E. coli* in human blood [19]. The following genes were identified in our primary screen: *guaB*, inosine monophosphate dehydrogenase involved in guanosine monophosphate biosynthesis; *ntpA*, dATP pyrophosphohydrolase involved in degradation of dATP; *pyrC*, dihydroorotase catalyzes the conversion of carbamoylaspartate to dihydrooratate; and *yeiA*, dihydropyrimidine dehydrogenase catalyzes first step in degradation of uracil and thymine (Table S2).

### Genes encoding surface structures

Several genes encoding surface structures that could potentially be involved in direct interaction with host cells were identified in our screen (Table S2). Periplasmic murein-peptide binding protein precursor gene (*mppA*) and a periplasmic protease that processes penicillin-binding protein 3 (*prc, c2239*) [20] are peptidoglycan biosynthetic genes that were identified in our primary screen. *arnT* and *yfbH* genes involved in resistance to polymyxin B, a peptide antibiotic that mimics the activity of host-derived cationic antimicrobial peptides, were identified in our primary screen. ArnT reduces the negative charge on the lipopolysaccharide due to its 4-amino-4-deoxy-L-arabinose transferase activity [21]. YfbH is a homolog of PmrJ, a deacetylase involved in biosynthesis of amino-arabinose-modified lipid A [22]. Two outer membrane porins, *ompC* and *ompG* were also identified in our screen. Colanic acid is a surface polysaccharide that is associated with biofilm formation in *E. coli* [23]. Two genes involved in colonic acid biosynthesis, *wcaM* and *wcaL*, [24] were identified as candidate fitness genes in the mouse bacteremia model. *pgaABD*, *c2394-95* and *yddB* are other genes associated with surface structures identified in our screen and were subjected to further investigation.

### Iron acquisition genes

Mammalian hosts actively limit the bioavailability of iron to hamper the growth of invading pathogens. Multiple genes involved in distinct iron acquisition systems were identified in our screen (Table S2). The *sitC* gene harbored on a bacteriophage, part of the SitABCD system involved in manganese and iron transport [25], was among the candidate fitness genes identified in this study (Table S2). A *chuA hma* double mutant, lacking two heme receptors, was previously found to exhibit fitness defect during bacteremia [11]. In the current study, mutation in *hma* alone reveals a fitness defect (Table S2) suggesting that heme is a major source for iron during systemic infection in a mammalian host. Multiple insertion sites were found within the genes involved in enterobactin biosynthesis, export, uptake and utilization with a mean fitness factor of 3.2. Salmochelin is a glycosylated derivative of enterobactin that evades chelation by host protein lipocalin-2 [26]. Inactivation of genes involved in salmochelin biosynthesis (*iroB*) and uptake (*iroN*) also resulted in attenuated fitness (Table S2). Insertion mutants in yersiniabactin biosynthesis and uptake genes also revealed a minor fitness defect (Table S2). Yersiniabactin, however, is not produced by *E. coli* CFT073 due to a previous insertion event at this locus. Coprogen and hydroxamate siderophore receptor gene, *fhuE*, was found among the candidate fitness genes (Table S2). Our results are consistent with the previously established role of iron acquisition genes in fitness of UPEC during systemic infection.

### Co-infection experiments validate the findings of primary screen

Tn-insertions leading to fitness defect in multiple genes within an operon/cluster and genes that were previously not known to affect fitness of UPEC during systemic infection were selected for further validation. Additionally, we tested the role of *pic* and *vat* in fitness primarily to establish that we have utilized a conservative threshold to delineate fitness genes. Co-infection experiments were performed by inoculating mice intravenously with equal numbers of both wild-type and mutant bacteria lacking select genes identified in the primary screen. Since most cases of bacteremia caused by UPEC are a result of ascending UTIs, we also tested the ability of a subset of these mutants to colonize murine urinary tract.

**Figure 3:**
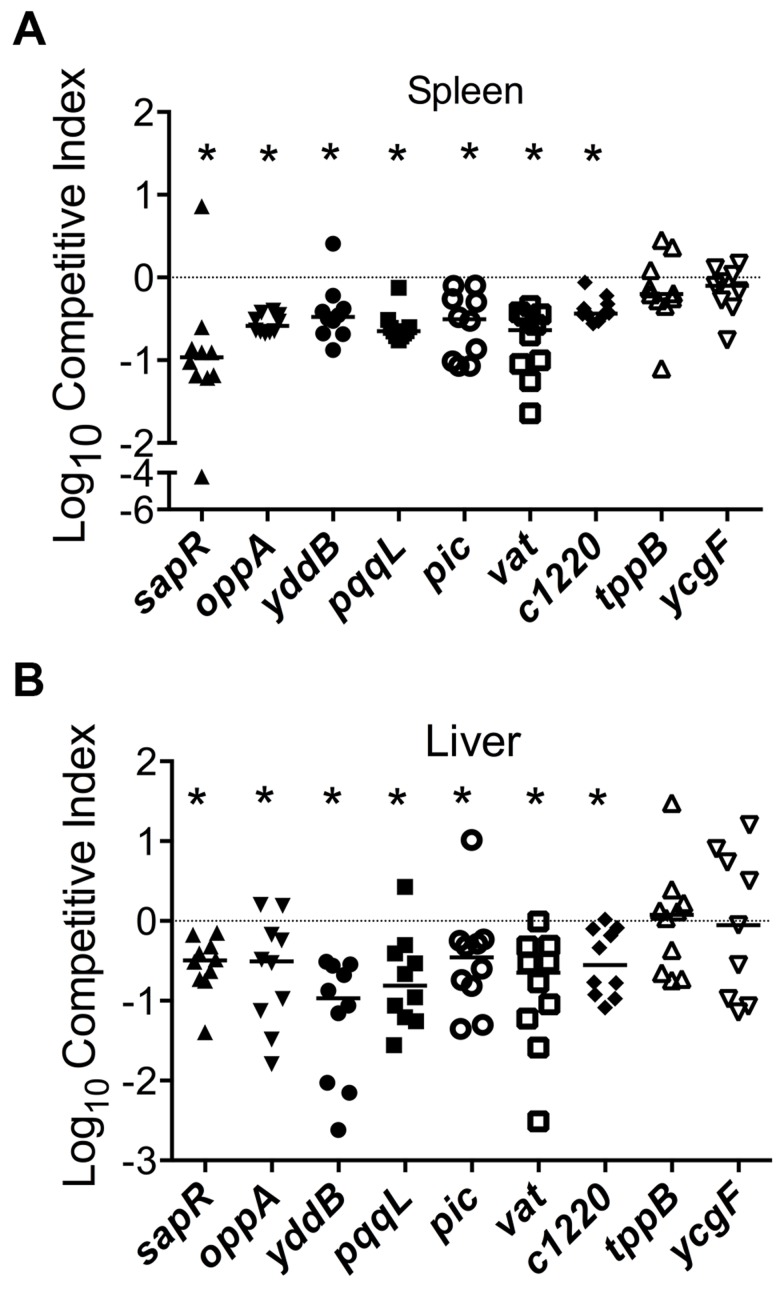
Validation of fitness genes identified in the primary screen. Mice were inoculated with an equal number of wild-type and mutant strain via the tail vein (n = 10). Spleen (A) and liver (B) were collected 24 hpi and plate counts were used to determine the competitive fitness of select mutants identified in our primary screen. Values less than 0 indicate reduced fitness compared to the parent strain. *P<0.05, Wilcoxon signed-rank test. *sapR*, sensitive to antimicrobial peptide gene cluster; *oppA*, oligopeptide uptake periplasmic binding protein; *yddB*, putative outer membrane receptor; *pqqL*, putative periplasmic zinc metallopeptidase; *pic*, protease involved in colonization; *vat*, vacuolating autotransporter toxin; *c1220*, 3-deoxy-D-*arabino*-heptulosonic acid-7-phosphate synthase; *tppB*, tripeptide uptake protein; and *ycgF*, an EAL domain protein.

**Figure 4:**
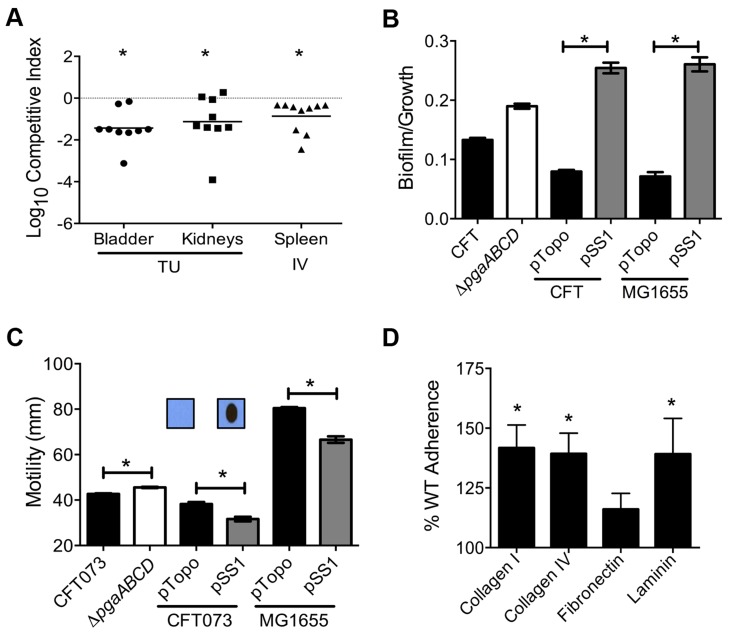
Poly-N-acetyl glucosamine is a fitness factor that promotes UPEC biofilm formation. (A) A mutant lacking the *pgaABCD* operon exhibited reduced fitness in the urinary bladder, kidneys (transurethral infection, n = 9) and spleen (intravenous infection, n = 10), compared to the wild-type strain, in a murine model of ascending UTI and systemic infection, respectively (*P<0.05, Wilcoxon-signed rank test). TU, transurethral; and IV, intravenous (tail vein). (B) PNAG promotes biofilm formation in UPEC upon overexpression. Crystal violet binding assay was used and optical density was normalized to growth. PNAG enhances biofilm formation in uropathogenic *E. coli* CFT073 and MG1655 genetic backgrounds. (C) PNAG production is inversely related to motility in *E. coli*. Immunoblots with anti-PNAG antibody are depicted (inset) and reveal overexpression of PNAG from pSS1. Deletion of the *pgaABCD* operon promotes swimming motility and overexpression reduces motility compared to vector control in both CFT073 and MG1655 genetic backgrounds. (D) PNAG mediates adhesion to extracellular matrix proteins. Percent adherence of the PNAG overexpression strain (>100%) was compared to vector alone control (100%). CFT073, wild-type; Δ*pgaABCD*, PNAG biosynthetic mutant; pTopo, pCR2.1 TOPO vector; pSS1, pCR2.1 TOPO+*pgaABCD* operon; and MG1655, a laboratory adapted K-12 strain. *P<0.05, Bonferroni's multiple comparison test following ANOVA.

**Figure 5:**
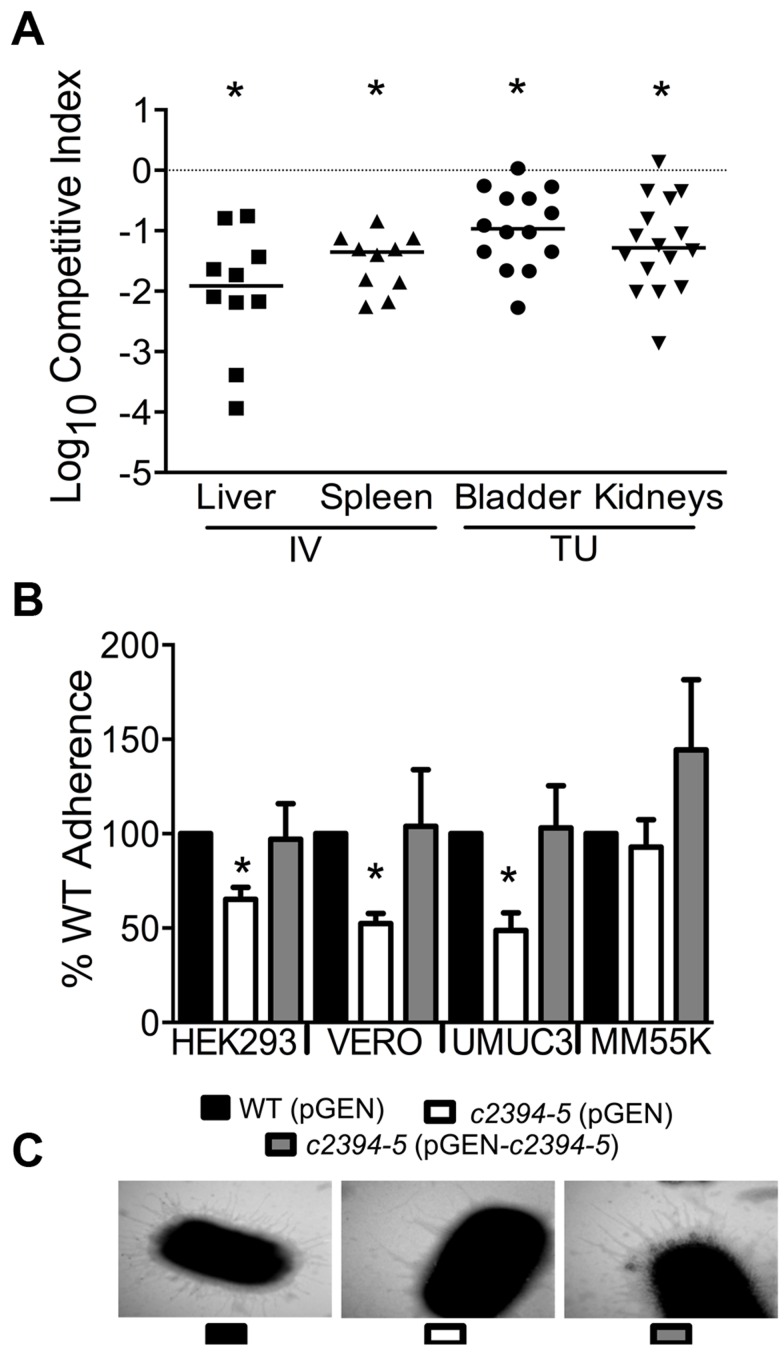
Putative type IV pilus subunit genes are involved in fitness in CFT073. (A) Competitive indices were determined from co-challenge infections with CFT073 and a mutant lacking major and minor type IV pilin genes (*c2394-95*). Fitness was determined during both systemic infection and UTI and each data point corresponds to results from an individual mouse (*P<0.05, Wilcoxon-signed rank test). IV, intravenous (tail vein); and TU, transurethral. (B) Loss of putative type IV pilin subunits results in reduced adherence to human and monkey kidney cells and human bladder cells. HEK293, human embryonic kidney; VERO, African green monkey kidney; UMUC3, human bladder; MM55K, murine kidney; WT (pGEN), wild-type with empty vector; Δ*c2394-95* (pGEN), mutant with empty vector; Δ*c2394-95* (pGEN-*c2394-95*), complemented mutant. *P<0.05, *t* test. (C) Electron micrograph of wild-type, mutant and complemented mutant strains. Wild-type and complemented mutant strains express more pili compared to the mutant strain (46,000 X magnification).

Growth kinetics of all the mutants used in the experiments described in the following sections is indistinguishable from that of the wild-type strain (Fig. S1). Homogenates of organs (spleen and liver from bacteremia model; urinary bladder and kidneys from ascending UTI model) were plated on plain and selective media. Differential plate counts were used to determine bacterial loads of wild-type and mutant strain in each tissue. Competitive indices (CI) were calculated using colony counts as: [mutant CFU/wild-type CFU (output)]/[mutant CFU/wild-type CFU (input)]. CI values less than 0 (log_10_ scale) indicate a comparative fitness defect for the mutant with respect to wild-type strain (Fig. 3A and 3B, 4A and 5A). Nine of the 11 mutants (82%) were out-competed by the wild-type strain during co-infection indicating that a high proportion of the candidate fitness genes identified in the primary screen indeed function as fitness factors during systemic infection (Fig. 3A and 3B, 4A and 5A). After successful validation of the primary screen, we probed the function of select candidate fitness genes in UPEC.

### *pgaABCD* operon is required for fitness in a mouse model of bacteremia and UTI

Transposon insertions within *pgaA*, *pgaB*, and *pgaD* resulted in reduced fitness, corresponding to fitness factors of 7.43, 9.41, and 4.32, respectively (Tables 1 and S2). The *pgaABCD* operon is involved in the biosynthesis and export of an extracellular polysaccharide, poly-N-acetyl glucosamine (PNAG), in *E. coli* [27]. Loss of PNAG biosynthetic operon resulted in a fitness defect in a mouse model of bacteremia (spleen, *P* = 0.002; Fig. 4A). The *pgaABCD* mutant was out-competed by the wild-type strain, ∼10-fold, both within the urinary bladder and the kidneys demonstrating that PNAG acts as a fitness factor *in vivo* within the murine urinary tract (Fig. 4A). The *pgaA* gene was upregulated 32-fold and *pgaC* transcript was detected by RT-PCR in urine collected from mice infected with *E. coli* CFT073, indicating that these genes are highly expressed during UTI. Furthermore, transcriptome analysis of UPEC CFT073 revealed that the *pgaABCD* genes are upregulated (∼2-fold) during culture in human urine compared to LB (unpublished results).

### PNAG promotes biofilm formation in UPEC

Since PNAG is involved in biofilm formation in a non-pathogenic strain of *E. coli* [27], we tested the contribution of PNAG to biofilm formation in the UPEC strain CFT073. Biofilm-forming ability of wild-type and *pgaABCD* mutant was tested using a crystal violet binding assay. Loss of PNAG did not affect biofilm formation on polystyrene (Fig. 4B) or glass surface (data not shown). Since UPEC is decorated with several surface structures, including multiple fimbriae and autotransporter adhesins, which might compensate for the loss of PNAG-dependent adhesion, the effect of overexpression of the *pgaABCD* operon on biofilm formation was also tested. Full-length *pgaABCD* operon including the native promoter was cloned into a multi-copy vector (pSS1); PNAG could be readily detected, by immunoblot analysis, in the overexpression strain but not in the vector control (Fig. 4C inset). Upon overexpression, PNAG promotes robust biofilm formation in UPEC strain CFT073 (Fig. 4B). *E. coli* K-12 strain MG1655 also displayed a profound, PNAG-dependent increase in biofilm formation, indicating that PNAG promotes biofilm formation in *E. coli* using a non strain-specific mechanism (Fig. 4B).

### Production of PNAG is inversely related to motility

Factors involved in adherence are known to affect motility in UPEC [28]. In strain CFT073, loss of PNAG production results in a significant increase in motility (Fig. 4C) that is accompanied by a 4-fold increase in the expression of *fliC* (data not shown). A higher level of *fliC* expression (encoding flagellin, the major structural subunit of flagella) explains the increased motility observed in the *pgaABCD* mutant. Conversely, overexpression of PNAG diminishes motility (Fig. 4C) suggesting that PNAG production and motility could be controlled in a reciprocal manner. Overexpression of PNAG also resulted in decreased motility in *E. coli* K-12 (Fig. 4C). Taken together, motility is adversely affected during PNAG overexpression in a non strain-specific manner. Additionally, known repressors of flagellar motility, PapX and FocX, are not involved in this crosstalk between PNAG levels and swimming motility (data not shown).

### PNAG affects adhesion to extracellular matrix (ECM) proteins

Intact epithelial surface precludes the access of pathogens to ECM proteins; however, inflammation-associated mucosal denudation results in contact with ECM proteins. A plate-based adherence assay was used to determine whether PNAG is involved in adherence to common ECM proteins collagen I, collagen IV, fibronectin and laminin. PNAG overexpression resulted in significantly higher adherence of UPEC strain CFT073 to collagen I, collagen IV and laminin (Fig. 4D) compared to vector control. PNAG does not affect binding to fibronectin under the assay conditions tested. To determine if PNAG protects UPEC from killing by macrophages, survival of wild-type, *pgaABCD*, wild-type (pSS1), and wild-type (pTopo) within the murine macrophage cell line RAW264.7 was assessed. Under our experimental conditions, PNAG did not contribute to adherence or intracellular survival (data not shown).

### Components of a putative type IV pilus facilitate colonization by UPEC

*c2394* encoding PilV was identified in the primary screen as a putative fitness gene (fitness factor = 6.7, Table S2). *pilV* (*c2394*) and *pilS* (*c2395*), encoding pilin subunits of type IV pilus two, are highly associated with UPEC strains compared to fecal *E. coli* isolates [29]. Additionally, these genes are more prevalent in *E. coli* isolated from humans than from animals [29]. Co-infection experiments revealed that the mutant strain lacking *pilV* and *pilS* genes was significantly out-competed (*P*<0.05) by wild-type *E. coli* CFT073 in the bladder, kidneys, spleen and liver (Fig. 5A). Our data demonstrate that these putative type IV pilin subunit genes are involved in colonization during both systemic infection and UTIs.

### *pilVS* are required for wild-type level adherence to uroepithelial cells

The ability of the isogenic mutant, *c2394-95* (pGEN), and the complemented strain, *c2394-95* (pGEN- *c2394-95*) to adhere to the immortalized epithelial cell lines UMUC-3 (human bladder), HEK293 (human embryonic kidney), VERO (green monkey kidney), and MM55K (mouse kidney) was compared to that of wild-type (pGEN) strain. Compared to wild-type, *c2394-95* mutant was less adherent to UM-UC-3 (*P* = 0.032), HEK293 (*P* = 0.031), and VERO (*P* = 0.012) (Fig. 5B) cells. However, no significant difference was observed on MM55K (*P* = 0.675) cells. Complementation restored adherence to wild-type levels on all cell lines (Fig. 5B). This suggests that *c2394-95* encode proteins involved in adherence to uroepithelial cells and the receptor for type IV pilin is likely expressed by both bladder (human) and kidney (human and monkey) epithelial cells, but not by the mouse kidney cell line. Electron microscopy was used to determine if the type IV pilus is indeed found on the cell surface. Wild-type and complemented mutant cells are densely piliated compared to the mutant strain that is sparsely piliated (Fig. 5C).

### *pilVS* are required for wild-type levels of swimming motility

A *c2394-95* mutant had a swimming diameter of 44.9±7.7 mm, significantly lower than that of wild-type *E. coli* CFT073 (*P* = 0.005), which swam 59.8±4.3 mm. Motility was not restored to wild-type levels by complementation (38.4±6.0 mm) with *c2394-95 in trans*; instead, the motility defect was increased upon expression of the pilin genes, which suggests that there may be a decrease in motility due to the level of expression from a multi-copy plasmid. Deletion of *c2394-95* does not appear to affect cell aggregation, biofilm formation or invasion of kidney epithelial cells in *E. coli* CFT073 (data not shown).

### Peptide uptake systems are involved in the fitness of UPEC

Multiple peptide uptake genes (*oppABD*, *sapACF* and *tppB*) were identified as candidate fitness genes in the primary screen (Tables 1 and S2). Mutants lacking the *sap* gene cluster or *oppA*, but not the *tppB* were found to exhibit fitness defects in spleen and liver during co-challenge experiments, compared to the wild-type strain (Fig. 3). The *opp* gene cluster harbors the genes involved in oligopeptide uptake and multiple transposon insertion sites were observed within these genes (Table S2). This observation suggests that the ability to utilize oligopeptides as a source of carbon and nitrogen is critical for UPEC survival in murine spleens. Transposon insertions and corresponding fitness factors for the *sap* genes are depicted in Fig. 2A.

**Figure 6:**
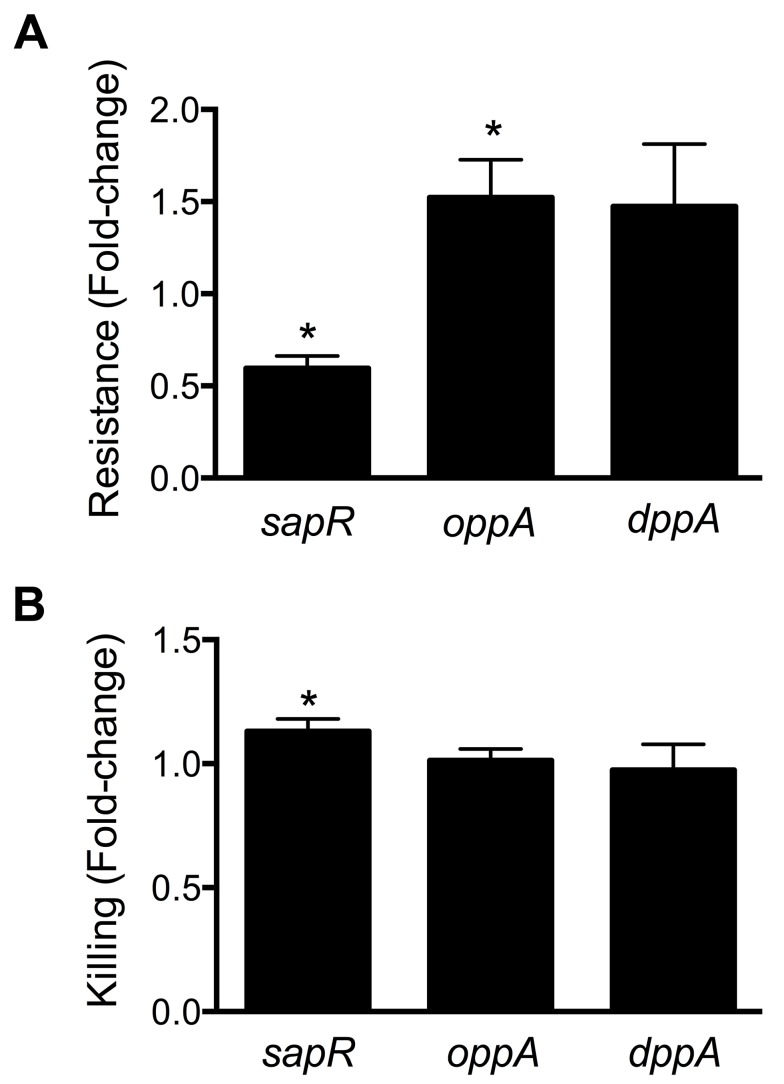
Involvement of peptide uptake system genes in resistance to polymyxin B and survival within macrophages. (A) *sap* genes are involved in protection against antimicrobial peptides. Polymyxin B was used to mimic host-derived cationic antimicrobial peptides and fold-change in percent resistance compared to wild-type is presented here. (B) Killing of peptide uptake mutants within the murine macrophage cells (RAW 264.7). Percent killing of intracellular bacteria was determined by gentamicin protection assay and fold-change in killing, relative to wild-type is depicted. *P<0.05, *t* test.

Cationic antimicrobial peptides represent a major antimicrobial defense system that aids in clearing invading pathogens. Polymyxin B (PB) is a peptide antibiotic that emulates the activity of host-derived cationic antimicrobial peptides [30]. The role of peptide uptake systems in resistance of UPEC to PB was tested. A mutant defective in dipeptide uptake (*dppA*) [31], not identified in our primary screen, was also used to determine if multiple peptide uptake systems are involved in PB resistance. Bacterial cultures in exponential phase of growth were exposed to PB and percent survival was calculated using colony counts from PB-treated and control cultures. Fold-change in resistance was calculated as the ratio of survival percentage of a given mutant to that of wild-type strain (Fig. 6A). Compared to the wild-type strain, the *sapR* mutant, that lacks the *sapABCDF* genes, exhibited increased sensitivity to PB (Fig. 6A, *P*<0.0001). The *oppA* mutant exhibited decreased sensitivity to PB compared to wild-type strain (Fig. 6A, *P* = 0.03) and the *dppA* mutant also showed a trend towards decreased sensitivity to PB (Fig. 6A).

Gentamicin protection assay was used to determine if the peptide uptake systems contributed to intracellular survival of UPEC in murine macrophage cells (RAW 264.7). Plate counts were used to determine the number of bacteria that entered and survived within RAW264.7 cells for 2 h. Ratio of killing percentages were determined and values >1 indicate that a given mutant was defective in intracellular survival within RAW 264.7 cells (Fig. 6B). A modest, but statistically significant reduction (*P* = 0.03) in intracellular fitness of the *sapR* mutant was observed (Fig. 6B), whereas the *oppA* and *dppA* mutants were not defective in intracellular survival compared to wild-type strain.

Although *tppB* was identified as a fitness gene in the primary screen, a co-challenge experiment revealed no role for this gene in fitness (Fig. 3). This discrepancy could be due to the differences in the nature of competition during infection with the transposon mutant library in the primary screen *versus* one-to-one competition between wild-type and mutant strains in our secondary validation experiments.

### *yddABpqqL* gene cluster augments fitness during systemic infection

**Figure 7:**
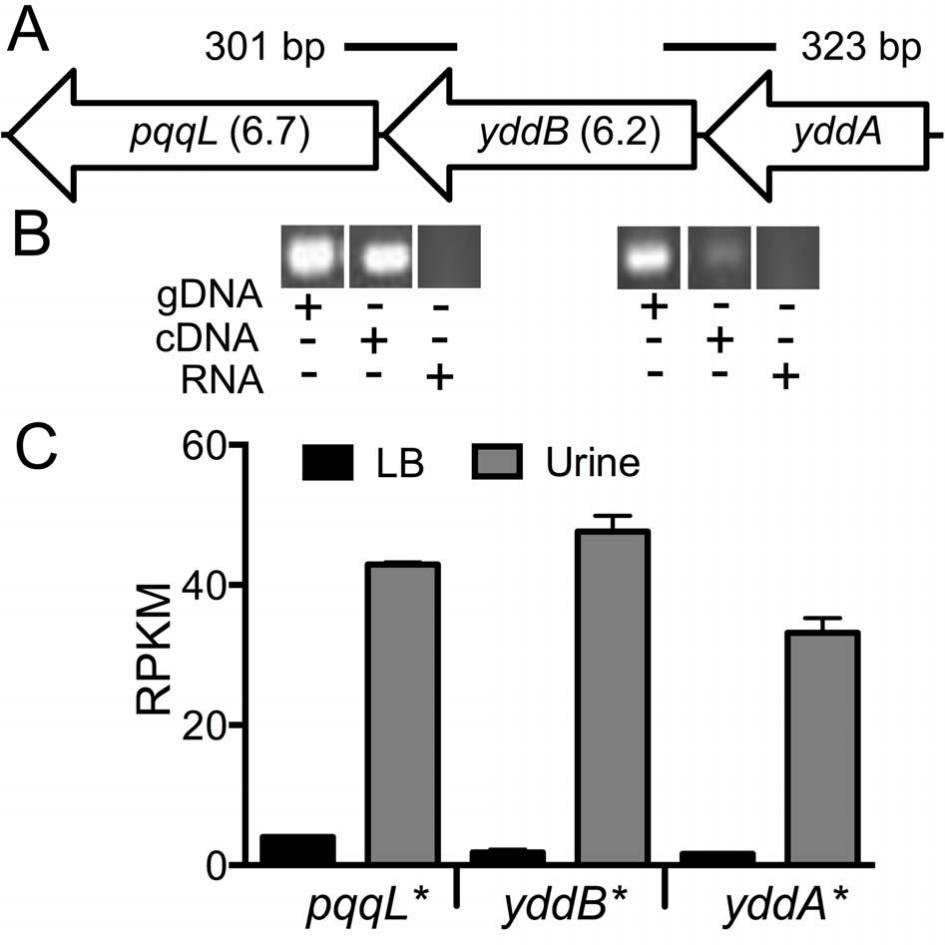
*yddABpqqL* genes are up-regulated during growth in human urine. (A) The *yddABpqqL* locus is depicted along with the median fitness factors (in parentheses) for the transposon insertion mutants. Solid lines indicate the regions amplified by PCR in B. (B) Co-transcription of *yddABpqqL* genes. RT-PCR reveals ∼300 bp amplicons that span adjacent genes. gDNA and RNA were used as positive and negative controls, respectively. (C) Gene expression in human urine. Gene expression was quantified by RNAseq in wild-type bacteria cultured *in vitro*. All three genes are highly upregulated (>30-fold, *P<0.05, Bonferroni's multiple comparison test following ANOVA) during growth in urine compared to LB. LB, lysogeny broth; and RPKM, reads per kilobase per million mapped reads.

In the *E. coli* CFT073 genome, *yddA*, *yddB* and *pqqL* encode an ABC transporter ATPase, an outer membrane β-barrel protein and an inner membrane-associated zinc metallopeptidase, respectively. *yddB* and *pqqL* were identified as fitness genes in our primary screen and median fitness factors for multiple insertion mutants in these genes are depicted in Fig. 7A. A BLAST search revealed that this gene cluster is found only among *E. coli* and *Shigella* strains. *yddB* and *pqqL* genes are involved in fitness during systemic infection in a mammalian host (Fig. 3). RT-PCR experiments revealed that these genes are indeed co-transcribed as a single mRNA (Fig. 7B).

YddB exhibits a high degree of sequence similarity to ligand-gated outer membrane β-barrel proteins such as ferrienterobactin receptor, FepA in *E. coli*. Since outer membrane β-barrel proteins are usually involved in iron uptake and a putative Fur box (GGGAATGGTTATCATTAG) is found overlapping the start codon of *yddA*, we tested whether these genes are differentially expressed during culture in human urine, an iron limited milieu. RNA was extracted from CFT073 bacterial cells cultured to mid-exponential phase in either LB or filter-sterilized human urine and gene expression was quantified using RNAseq (unpublished results). The *yddA*, *yddB*, and *pqqL* genes are highly upregulated (>30-fold) during growth in human urine compared to LB (Fig. 7C). We also tested whether iron levels directly regulate the expression of *yddA-yddB-pqqL* genes. Transcript levels were determined in wild-type strain cultured in LB, LB supplemented with an iron chelator (Dipyridyl) or additional iron. Iron levels alone do not affect the expression of these genes in UPEC (Fig. S2A). However, *yddA*, *yddB*, and *pqqL* genes are upregulated in the Δ*fur* mutant that lacks ferric uptake regulator (Fur), compared to the wild-type strain (Fig. S2B). Taken together, our data indicate that these genes are upregulated during growth in human urine but not subject to regulation by iron levels alone.

### Autotransporter toxins Pic and Vat

Proteins in the SPATE (serine protease autotransporter proteins of *Enterobacteriaceae*) family have previously been implicated in the pathobiology of UPEC [32]. Genes encoding members of SPATE family, protease involved in colonization (Pic) and vacuolating autotransporter toxin (Vat, previously known as Tsh) were identified in our primary screen (Table S2). The *pic* and *vat* transposon insertion mutants exhibited a fitness factor of 5.2 and 4.4, respectively. Co-infection experiments were performed with these genes to test whether a conservative threshold was used to delineate fitness genes. Competitive indices reveal that both *pic* and *vat*, which exhibit fitness factors lower than the cutoff used to delineate fitness genes, play a role in the fitness of UPEC during systemic infection in mice (Fig. 3).

### *c1220*, a shikimate pathway gene

3-deoxy-D-*arabino*-heptulosonic acid-7-phosphate synthase (DAHPS), encoded by *c1220*, catalyzes the formation of 3-deoxy-D-*arabino*-heptulosonate 7-phosphate (DAHP) from phosphoenolpyruvate and erythrose 4-phosphate, an early step in shikimate biosynthesis [33]. In CFT073, *c1220* is located on the *serX* pathogenicity island [34]. DAHPS encoded by *c1220* is the fourth isozyme, in addition to *aroF*, *aroG* and *aroH* that catalyzes the production of DAHP in *E. coli* CFT073. Two transposon insertion sites mapped to this gene resulted in reduced fitness during survival in spleen. Co-infection experiments with a *c1220* mutant and wild-type strain confirmed that the mutant has a fitness defect in spleen and liver during systemic infection in mice (Fig. 3).

### YcgF, an EAL domain protein

A gene encoding an EAL domain protein, *ycgF*, was identified in our primary screen. EAL domain proteins are usually associated with phosphodiesterase activity that reduces the intracellular levels of an important intracellular messenger, cyclic-di-GMP [35]. YcgF has been designated as an inactive phosphodiesterase that nevertheless positively regulates swimming motility by increasing flagellin levels in CFT073 [35]. Although *ycgF* was identified as a candidate fitness gene, co-infection experiments failed to reveal a role for *ycgF* in fitness in a mouse model of systemic infection (Fig. 3).

## Discussion

Uropathogenic *Escherichia coli* (UPEC) is a major cause of bacteremia in humans, yet, there is limited understanding of the fitness mechanisms used by this important pathogen during bacteremia and systemic infection. Here, we describe screening transposon mutants of *E. coli* CFT073 in a murine model of systemic disseminated infection and identifying 242 candidate fitness genes. Specific mutations were introduced in 11 candidate fitness genes and the contribution of the following nine gene or gene clusters in fitness was confirmed: *pgaABCD* (biosynthesis and export of poly-N-acetyl glucosamine), *c2394-95* (major and minor pilin of type IV pilus two), *oppA* (oligopeptide uptake periplasmic-binding protein), *sapABCDF* (sensitive to antimicrobial peptide), *yddB* (putative outer membrane receptor), *pqqL* (zinc metallopeptidase), *c1220* (a shikimate pathway gene), and *pic* and *vat* (autotransporter serine proteases). 82% of the specific mutants in representative candidate fitness genes were significantly outcompeted by the wild-type strain, validating the TraDIS approach in our murine model of systemic infection.

Transposon mutagenesis has been an indispensable tool in unraveling gene function. The complex nature of experiments involving transposon mutant pools, including bottlenecks when screening signature-tagged mutants in animal models of infection [36], has resulted in screens with fewer mutants than required to achieve genome saturation. Recently, HT sequencing and bioinformatic analyses have been used in tandem to identify transposon insertion sites in genome-saturating transposon mutant pools [6]. Several variants of this approach include HITS, high-throughput insertion tracking by deep sequencing [37]; INSeq, insertion sequencing [38]; Tn-Seq, transposon sequencing [39]; and TraDIS, transposon directed insertion-site sequencing [7]. These techniques utilize chromosomal regions flanking the transposons as unique tags in lieu of the synthetic tags used in signature-tagged mutagenesis. Availability of a large number of bacterial genome sequences combined with cost-effective HT sequencing is poised to make these approaches a staple of functional genomic studies in the near future.

Understanding the fitness strategies employed by UPEC during infection of a mammalian host has the potential to identify targets for novel intervention strategies. Here, we describe the first comprehensive identification of fitness factors involved in systemic infection by an ExPEC strain, CFT073, in a mammalian host. The original work describing TraDIS catalogued the essential genes in *Salmonella enterica* subsp. *enterica* serovar Typhi [7]. Since the primary objective of our study was to identify *in vivo* fitness factors, the mutant pool was passaged in LB to deplete mutants with *in vitro* fitness defects from the inoculum. This might have led to a reduction in the diversity of the mutant pool used for infection, compared to the original pool comprising of 360,000 transformants and may explain the fact that we identified only 6732 independent transposon insertion sites. Notwithstanding the reduced complexity of the input pool, we have identified novel fitness factors from this study.

TFAST was previously developed in our laboratory to determine the transcription factor binding sites [17] and facilitated successful identification of PapX binding site in the *flhDC* promoter [40]. Here, TFAST was applied to determine the chromosomal location and the frequency of detection of a given transposon mutant. Potential fitness genes identified in this study could be an underestimate because genes *pic* and *vat* did not meet the threshold (mean+2 standard deviations) but were confirmed as fitness genes in the co-infection experiments. The EZ-Tn*5* transposon used for random mutagenesis was not modified to incorporate promoter regions at either end; therefore, the transposon insertion mutations could exert polar effects on co-transcribed genes. Additionally, random events could result in the loss of a transposon mutant during infection and could result in misinterpretation as a fitness gene. In co-infection experiments, nine of the 11 (82%) tested mutants revealed a fitness defect confirming the validity of our primary screen.

Seven of 372 predicted transposon mutants with a fitness defect (1.9%) were found in another study as essential genes in a laboratory strain of *E. coli* [18]. Studies on gene essentiality in *E. coli* have been conducted primarily on non-pathogenic, laboratory-adapted strains. Genomes of UPEC are usually larger than these laboratory strains. For instance, genome of UPEC CFT073 is ∼590 Kbp longer than the widely studied *E. coli* K-12 strain MG1655 [14]. Depending on the transposon insertion site and growth conditions, it is possible that transposon insertions could be tolerated in some essential genes. For instance, *degS* is designated as an essential gene in *E. coli* [18]. However, a *degS* mutant has been successfully constructed in *E. coli* CFT073 and used to demonstrate that DegS, likely by modulating members of Sigma E regulon, affects the fitness of UPEC during peritonitis as well as during UTI in a mouse model of infection [41]. Another possible explanation is the emergence of suppressor mutations that negate the effects of original mutation. It is also possible that these are artifacts due to sequence similarity to parts of other non-essential genes or gene duplication events. Potentially, some of the essential genes involved in non-structural components could be complemented by other transformants within the mutant pool. These genes constitute only a small fraction of all the fitness genes unraveled in this study.

A previous study in ExPEC during systemic infection in a mammalian host, led to the identification of type 1 pilus; P fimbria; Hma and ChuA, heme receptors; TonB, iron uptake energy transducer; Ksl, K2 capsule biogenesis; and NanA, *N*-acetylneuraminate aldolase as fitness determinants [11]. This study has greatly expanded the potential bacteremia fitness determinants in UPEC and offers evidence for the role of nine of these novel fitness determinants in a murine model of systemic infection. Furthermore, 81 (33.5%) of the candidate fitness genes are predicted to encode hypothetical proteins and constitute a unique resource that can be exploited to identify previously unknown fitness determinants.

Biosynthetic mutants defective in either salmochelin or both salmochelin and enterobactin production revealed reduced fitness in the primary screen. Since these mutants retain the ability to utilize catecholate siderophores synthesized by other transformants, it is likely that the observed fitness defect emerges from iron uptake-independent roles. Recently, catecholate siderophore biosynthesis, but not uptake-alone, was demonstrated to mitigate the effects of oxidative stress in both *Salmonella* Typhimurium and *E. coli* [42]. It is, therefore, plausible that UPEC utilizes catecholate siderophore biosynthesis not only for canonical iron acquisition functions but also for protection against oxidative stress encountered during systemic infection.

PNAG, an extracellular polysaccharide, has been associated with the virulence in a broad spectrum of bacterial pathogens, including *Aggregatibacter actinomycetemcomitans* [43], *Bordetella pertussis* [44], *Staphylococcus aureus* [45], and *Yersinia pestis* [46]. Antibodies raised against *S. aureus*-derived PNAG confer passive protection against systemic infection with a clinical UPEC strain [47]. Here, we provide evidence that biosynthesis of PNAG is required for optimal fitness of UPEC during both UTI and systemic infection (Fig. 4).

Type IV pili are filamentous organelles found at the bacterial surface that affect adherence and motility in several bacterial species, including enteropathogenic *E. coli* [48]. We found that the genes encoding predicted major and minor type IV pilins (*c2394-95*) are critical for fitness during both bacteremia and UTIs. Although the mutant did not exhibit reduced adherence to MM55K cells, an immortalized cell line derived from the kidneys of AKR strain mice, the mutant revealed colonization defect in murine kidneys in a mouse model of ascending UTI. Differences in the expression of surface receptors on MM55K cells compared to those found within the nephrons of live CBA/J mice used for infection experiments could account for the discrepancy between adherence phenotypes observed for the type IV pilus mutant during *in vitro* and *in vivo* assays. Electron micrographs revealed reduced number of pili in the mutant compared to wild-type and complemented mutant strain. However, UPEC strain CFT073 produces multiple fimbria [29]; therefore, this observation must be verified with immunostaining to enable specificity. UPEC also harbors a locus similar to that encoding type IV pilus in *E. coli* K-12 and has been demonstrated to affect the fitness in mouse urinary tract [49]. Mutants in both type IV pilus loci exhibit fitness defects independent of each other and here we demonstrate that type IV pilus two is a novel fitness factor in UPEC.

Oligopeptide uptake system gene *oppA* was previously shown to be critical for fitness of UPEC in the urinary tract [31]. Gene clusters involved in peptide uptake, *opp* and *sap*, were found to contribute to the fitness of UPEC during systemic invasion in the current study. The *sapABCDF* gene cluster contains homologs of genes involved in sensitivity to antimicrobial peptides in *Salmonella enterica* subspecies Typhimurium [50] and non-typeable *Haemophilus influenzae* [51]. Our data support a model in which the *sap* gene cluster, but neither *oppA* nor *dppA*, is required for optimal protection against polymyxin B and intracellular survival in murine macrophages (Fig. 6).

Targets of Fur in *E. coli* MC4100 were detected using a macroarray and *yddABpqqL* was determined as a Fur-regulated gene cluster in *E. coli* [52]. A transposon mutant screen in *E. coli* strain CC118, revealed that *yddA* and *yddB* are required for optimal growth in rich medium at 37°C [53]. However, UPEC CFT073 mutants lacking *yddB* or *pqqL* genes exhibited no difference in growth rate compared to wild-type strain *in vitro* (Fig. S1). *yddA* acts as a colonization factor in enterohemorrhagic *E. coli* O26:H^−^ in a calf model of intestinal colonization [54]. Enhanced expression in urine (Fig. 7C) and the high degree of identity of YddB protein to ligand-gated outer membrane siderophore receptors allowed us to speculate that these genes could be involved in iron uptake. Although these genes are regulated by Fur (Fig. S2B), they do not appear to be regulated by iron levels alone in CFT073 (Fig. S2A). Cues, other than reduced iron levels, sensed by UPEC in human urine likely govern the regulation of *yddABpqqL* genes. *pqqL* from *E. coli* has been previously shown to complement pyrolloquinoline quinone (PQQ) biosynthetic genes *pqqE* and *pqqF* in *Methylobacterium organophilum* [55]. PQQ is a cofactor for quinoproteins, including glucose dehydrogenase in *E. coli*. It must be noted that *E. coli* is not capable of PQQ biosynthesis [55]. Studies are in progress to address whether this gene cluster is involved in uptake and processing of PQQ.

We have identified Pic and Vat, autotransporter serine proteases, to be involved in fitness during bacteremia (Fig. 3). In *E. coli* CFT073, *pic* was upregulated during UTI in a murine host and Pic exhibited serine protease activity *in vitro* [32]. On the contrary, Vat (referred to as Tsh in [32]) did not exhibit a detectable serine protease activity and both of these genes did not appear to affect fitness of UPEC during UTI. Key human leukocyte adhesion molecules such as CD43, CD44, CD45 and CD93, are targeted by Pic resulting in deregulation of leukocyte migration and inflammation [56]. Therefore, it is possible that reduced fitness of Pic mutants during systemic infection could emerge from its role in modulating inflammatory response to systemic infection with UPEC.

Shikimate is a critical intermediary molecule in chorismate biosynthetic pathway and chorismate is a precursor for the biosynthesis of aromatic amino acids, catecholate siderophores, folate, menaquinone and ubiquinone in bacteria [33]. Biosynthesis of aromatic amino acids has been associated with virulence in several bacterial species in various animal models of infection, including *Neisseria meningitidis* [57], *Proteus mirabilis* [58], *Salmonella enterica* subspecies *enterica* serovar Typhimurium [36], and *Staphylococcus aureus* [59]. Taken together, these findings indicate that aromatic amino acids and other compounds derived from the chorismate pathway are critical for optimal fitness of multiple bacterial pathogens during infection.

In summary, a combination of transposon mutagenesis and HT sequencing was used to determine genes in UPEC that contribute to fitness in a mouse model of systemic infection. The role of multiple candidate fitness genes was confirmed by independent experiments using a mouse model of infection and *in vitro* assays. Further characterization of the fitness genes unraveled in this study has the potential to identify targets for developing novel intervention strategies against bacteremia caused by UPEC.

## Materials and Methods

### Ethics statement

All animal experiments were performed in accordance to the protocol (08999-3) approved by the University Committee on Use and Care of Animals at the University of Michigan. This protocol is in complete compliance with the guidelines for humane use and care of laboratory animals mandated by the National Institutes of Health.

### Bacterial strains and culture conditions

*E. coli* CFT073, a prototypical uropathogenic strain that caused bacteremia of urinary tract origin [12] was used to generate a saturating Tn*5* insertion mutant library. Strains and plasmids used in this study are listed in Table 2. Bacterial strains were cultured in LB containing 0.05% NaCl, unless otherwise noted. Tn*5* transformants were cultured in LB containing kanamycin (12.5 µg/ml). Lambda red recombineering was used to introduce specific mutations in strain CFT073 [60]. Genetically defined mutants used in this study were cultured in LB containing either kanamycin (25 µg/ml) or chloramphenicol (20 µg/ml). Oligonucleotide primers used in this study are listed in Table S1. Growth kinetics of the wild-type and mutant strains were determined using a Bioscreen C system (Growth Curves USA). Overnight cultures were diluted 1∶100 in LB and incubated at 37°C. Optical density values were recorded at 600 nm, every 15 min, for 22 h and included three biological replicates, comprised of two technical replicates.

**Table 2:** Bacterial strains and plasmids.

Strain	Description/Use^a^	Source
***E. coli***		
CFT073	Bacteremia isolate, wild-type	[12]
*pgaABCD*	CFT073 Δ*pgaABCD::cat*	This study
*yddB*	CFT073 Δ*yddB::npt*	This study
*pqqL*	CFT073 Δ*pqqL::npt*	This study
*oppA*	CFT073 Δ*oppA::npt*	[31]
*dppA*	CFT073 Δ*dppA::cat*	[31]
*c1220*	CFT073 Δ*c1220::npt*	A. Yep
*pic*	CFT073 Δ*pic::npt*	This study
*vat*	CFT073 Δ*vat::npt*	This study
*ycgF*	CFT073 Δ*ycgF::npt*	[35]
*tppB*	CFT073 Δ*tppB::npt*	This study
*c2394-95*	CFT073 Δ*c2394-95::cat*	This study
*sapR*	CFT073 Δ*sapR::npt*	This study
*fur*	CFT073 Δ*fur::npt*	A. Yep
**Plasmids**		
pKD46	Lambda red recombineering	[60]
pKD4	Template for *npt*	[60]
pKD3	Template for *cat*	[60]
pCR Topo 2.1	Multicopy vector	Invitrogen
pSS1	pCR Topo 2.1+*pgaABCD*	This study
pGEN	Low-copy number vector	[31]
pGEN- *c2394-95*	pGEN with *c2394-95*	This study

a *cat*, chloramphenicol acetyl transferase; *npt*, neomycin phosphotransferase.

### Generation of transposon mutants

Tn*5* insertion mutants were generated in *E. coli* CFT073 using the EZ-Tn*5* transposome kit (Epicentre). Briefly, transposome complexes were electroporated into *E. coli* CFT073 and bacteria were allowed to recover in SOC broth for 50 min prior to plating on LB agar containing kanamycin using an automated plater (Spiral Biotech). Plates were incubated overnight at 37°C and CFUs were enumerated using a Qcount colony counter (Spiral Biotech). A total of 360,000 transformants were generated for this study and archived in pools of 1800 CFUs. The entire Tn*5* mutant collection was passaged three times in LB for 16 h at 37°C and the resulting pool was used as the inoculum for experimental infections.

### Mouse infection experiments

CBA/J mice (6–7 week old, Harlan Laboratories) were inoculated with 10^6^ (n = 5), 10^7^ (n = 10) or 10^8^ (n = 5) CFU of CFT073 bacteria via tail vein. Mice were euthanized after 24 h and livers and spleens were harvested. Homogenates of these organs were plated on LB plates containing kanamycin and bacterial burden was determined. Mice were inoculated with 10^7^ (n = 20) CFU of CFT073::Tn*5* mutants. After 24 h, mice were euthanized to collect spleens. Homogenates of spleens were plated in their entirety, as described above and the bacterial burden was calculated. Colonies from splenic cultures were harvested and pooled from all 20 mice before archiving bacterial pellets at −80°C.

For co-infection experiments, wild-type and specific mutants, cultured overnight, were resuspended in PBS to yield 2×10^8^ CFU/ml, containing equal number of wild-type and mutant CFUs. Inoculum (100 µl) was administered via the tail vein and mice were euthanized 24 h pi. For the ascending UTI model, female mice were inoculated intravesically via a transurethral catheter with 50 µl of the inoculum containing 10^9^ CFU/ml (equal number of wild-type and mutant CFUs) and animals were euthanized after 48 hpi. Homogenates of spleen and liver (intravenous infection) or urinary bladder and kidneys (intravesical infection) were plated on plain and antibiotic-containing plates. Both wild-type and mutant strains grow on LB plates, whereas only a mutant strain can grow on antibiotic containing LB plates. Plate counts were used to calculate the number of wild-type and mutant bacteria surviving *in vivo*. Competitive indices (CI) were calculated as the ratio of mutant over wild-type in tissues to the ratio of mutant over wild-type in the inoculum. Urine was collected from a group of 5 mice infected with CFT073, periodically over 48 hours and immediately stabilized with RNAprotect (Qiagen) prior to RNA extraction.

### Illumina sequencing

Genomic DNA was isolated from the inoculum used for infections (input) and from cultures derived from infected spleens (output) using DNeasy blood and tissue DNA extraction kit (Qiagen). Genomic DNA (5 µg) was sheared to yield ∼300 bp fragments (Covaris). Illumina Truseq adapters were ligated to DNA fragments and used for Tn-specific amplification. A Tn-specific primer composed of the flowcell binding region of the Truseq adapter and Tn-specific region was used in conjunction with the Truseq adapter-specific primer to amplify transposon-chromosome insertion junctions (Table S1). Briefly, 25 ng of the TruSeq library was used as template for 30 cycles of amplification. Amplicons were further processed for Illumina sequencing (cluster generation) according to manufacturer's recommendations and sequenced using a Tn-specific primer (Table S1). Libraries from input and output samples were sequenced in two separate lanes of a single sequencing run in an Illumina HiSeq2000 sequencer. Library preparation and sequencing were performed at the University of Michigan DNA core facility.

### Mapping of transposon insertion sites

Reads from the input and output libraries, in FASTQ format, were aligned to the genome of *E. coli* CFT073 (NCBI accession no. NC_004431) [14] using the short read aligner BOWTIE [16]. The alignment files, in SAM format, were then used in the TFAST [17] program to determine the number of reads that map to a given chromosomal location in the input and output libraries.

### Crystal violet binding assay

To assess biofilm production, strains were cultured in tryptic soy broth containing 1% glucose in 96-well tissue culture-treated polystyrene plates for 24 h, at 37°C. Supernatants were aspirated and plates were washed three times with water and stained with 0.3% crystal violet solution for 10 min. Unbound crystal violet was removed by three additional washes with water. Biofilm-bound crystal violet was dissolved in 200 µl of 33% acetic acid and absorbance was measured at 590 nm (μQuant, Bio Tek instruments, Inc.). This experiment was repeated at least three times, independently.

### Detection of PNAG by immunoblotting

The protocol described by Cerca *et al*. [47] was adapted. Cultures, incubated overnight in tryptic soy broth containing 1% glucose, were adjusted to an OD_600_ of 1.5. Cell pellets from 1 ml samples were resuspended in 300 µL of 0.5M EDTA (pH 8.0) and boiled for 5 min. Samples were centrifuged at 13,000 RPM for 6 min. Supernatants were treated with Proteinase K (2 µg/µL), heat inactivated and diluted 3-fold in Tris-buffered saline (TBS; 20 mM Tris-HCl, 150 mM NaCl, pH 7.4). Extracts (200 µl/sample) were immobilized on nitrocellulose membranes and blocked with 5% skim milk in TBST (TBS containing 0.1% Tween20) for 2 h. Blots were incubated for 2 h with an affinity-purified anti *S. aureus* PNAG antibody (1∶2000) raised in rabbits [61]. Horseradish peroxidase-conjugated secondary anti-rabbit IgG antibody (1∶20,000) was used in conjunction with ECL Plus enhanced chemiluminescence detection system (GE Healthcare) to determine the presence of PNAG. These experiments were repeated at least three times, independently.

### Motility assays

Agar (0.25%) plates containing NaCl (0.5%) and tryptone (1%) were used to measure swimming motility. Ampicillin (100 µg/µl) was added for plasmid maintenance, when required. Cultures were stab-inoculated and incubated at 30°C for 16 h. Diameters (mm) of swimming zone were determined. Four independent experiments were performed with at least two technical replicates.

### Adherence to extracellular matrix proteins

*E. coli* CFT073 (pTopo) and CFT073 (pSS1) were cultured overnight in TSB with 1% glucose and ampicillin (100 µg/ml). Bacteria, washed and resuspended in PBS to an OD_600_ of 1, were incubated in ECM protein coated plates (Biocoat plates, Becton Dickinson) for 2 h at 37°C. The number of bacteria in the inoculum and the number of bacteria that remain in the supernatant (non-adherent) were determined by plate counts and used to calculate percentage of adherent bacteria. Fold-change in adherence was calculated as the ratio of adherence percentages of CFT073 (pSS1) over CFT073 (pTopo).

### Adherence to cells derived from urinary tract epithelium

The following immortalized cell lines were used in adherence assays: human bladder epithelium, UM-UC-3 (ATCC CRL-1749); murine kidney, MM55.K (ATCC CRL-6436); green monkey kidney, VERO (ATCC CCL-81); and human embryonic kidney, HEK293 (ATCC CRL-1573). Cells were cultured to confluence in 24-well cell culture plates (Corning) in Dulbecco's Modified Eagle Medium supplemented with 10% fetal bovine serum, penicillin (100 U/ml), streptomycin (100 µg/ml) and L-glutamine (0.3 mg/ml), referred to as DMEM-PSG, at 37°C in a humidified atmosphere with 5% CO_2_. Epithelial cell cultures were washed once with PBS, and inoculated with a 250 µl suspension containing 1×10^8^ CFU of *E. coli* CFT073 (wild-type), *c2394-95* mutant, or the complemented mutant in DMEM without antibiotics. Infected epithelial cells were incubated at 37°C with 5% CO_2_ for 30 min, and then washed three times with PBS. Epithelial cells, along with any adherent bacteria, were lifted by incubation in 1 ml sterile distilled water containing 5 mM EDTA. Colony counts were used to enumerate CFUs in the inocula and cell-associated bacteria. Adherence was expressed as cell-associated CFU/initial CFU. Adherence of each mutant was normalized to the wild-type control; assays were performed in triplicate, each with three technical replicates.

### Transmission electron microscopy

*E. coli* CFT073 (wild-type), *c2394-95* mutant, and the complemented mutant were cultured for 3 h at 37°C. Samples were swirled gently and 10 µl of the culture was dropped onto formvar carbon support film on TEM specimen grids (Electron Microscopy Sciences). Grids were incubated at room temperature for 5 min, and excess medium was wicked off with filter paper. Grids were washed once with 10 µl of deionized water, and then stained for 2 min with 10 µl of 1% phosphotungstic acid (pH 6.8). Excess stain was removed; grids were washed immediately with deionized water and dried. Grids were visualized using a Philips CM-100 transmission electron microscope.

### Polymyxin B sensitivity assays

Overnight cultures, diluted 1∶100 in fresh medium, were incubated at 37°C for 2 h. Cultures were exposed to polymyxin B (4 µg/mL) for 30 min. Colony counts were determined by plating and percent survival upon exposure to Polymyxin B was calculated as the ratio of CFU in the treated samples to untreated controls. The experiment was repeated at least three times, independently.

### Gentamicin protection assay

RAW 264.7 cells were cultured in RPMI1640-PSG supplemented with 10% fetal bovine serum and seeded in 24-well tissue culture plates. CFT073 and mutant strains, cultured overnight in LB, were washed in PBS and resuspended in RPMI1640 to an OD_600_ of 0.004. Cells were washed with PBS, overlaid with the inoculum at an MOI of 10 and incubated for 30 min. Two identical plates, for 0 h (T_0_) and 2 h (T_2_), were set up during each experiment. Supernatants were aspirated and cells were washed three times with PBS. Fresh RPMI1640 supplemented with gentamicin (200 µg/ml) was added and incubation was continued. The T_0_ plate was removed at 15 min post gentamicin addition and cells were lysed with saponin (10%, w/v in water). Lysates were plated to determine the number of intracellular bacteria. T2 plates were processed as described here at 2 h post gentamicin addition. Percent killing was calculated as the percent of intracellular bacteria that were killed within RAW 264.7 cells. Killing percentages of mutants were compared to that of wild-type bacteria to determine the comparative fitness of a given mutant during survival within RAW 264.7 cells. The experiment was repeated three times with three technical replicates per strain.

### RT-PCR

RNA was extracted from *E. coli* CFT073 cultured in LB or in filter sterilized human urine to mid-exponential phase or from cells harvested from urine of mice infected with wild-type strain using RNeasy mini kit (Qiagen). Contaminating DNA was removed by incubation with DNase (Turbo DNA-free, Ambion) and reverse transcribed using SS RT III (Invitrogen). To determine co-transcription, cDNA, genomic DNA and RNA samples were used as templates in standard PCR reaction (primers listed in Table S1). The entire experiment was repeated twice, independently.

Overnight cultures of *E. coli* CFT073 were diluted 1∶100 in LB or LB with 300 µM dipyridyl (Sigma) or LB with 10 µM ferric chloride and incubated for 2 h at 37°C. RNA extraction and cDNA synthesis were performed as described above. Expression of *yddA*, *yddB* and *pqqL* transcripts was determined by qPCR using SYBR green chemistry (Agilent Technologies) in a Stratagene Mx3000P thermal cycler (Stratagene). Transcripts were normalized to *gapA* mRNA (Table S1) and relative quantification was performed using CFT073 cultured in LB as the calibrator.

Overnight cultures of *E. coli* CFT073 and Δ*fur* mutant were diluted 1∶100 in LB and incubated for 2 h. RNA extraction, cDNA synthesis and qPCR were performed as described above. Relative quantification was performed using CFT073 as the calibrator. Both qPCR experiments were repeated three times with two technical replicates.

DNase-treated RNA from mouse UTI urine was used to determine levels of *pgaA* transcript by qPCR, essentially as described above. Mid-exponential phase cells from LB were used as calibrator and all samples were normalized to *gapA* levels.

### Statistical analyses

Statistical tests were performed using Prism 5 (www.graphpad.com). Data were analyzed using the following tests: co-infection experiments, Wilcoxon signed-rank test against a theoretical median of 0; biofilm assay, swimming motility assay and adherence to epithelial cells and ECM proteins, two-way ANOVA with Bonferroni's multiple comparison test; polymyxin B resistance assay and intracellular survival assay, student's *t* test. *P*<0.05 was considered as a statistically significant difference. Error bars in the figures represent standard error of the mean.

### Accession number

The raw reads can be accessed under the accession number, SRP027190 in NCBI SRA.

## Supporting Information

Figure S1 **Growth patterns of mutant strains are similar to wild-type strain.** Growth kinetics of wild-type and mutant strains, used in co-infection experiments, were determined in LB. Optical density measurements were recorded using a BioscreenC system. Differences in growth pattern are not discernible, indicating that these mutants are not compromised in fitness during growth in LB *in vitro*. Mean from three independent experiments are plotted here and error bars indicate SEM (not obvious in the figure due to small variation from the mean).
(TIFF)

Figure S2 **Iron levels do not regulate the expression of** ***yddA*** **,** ***yddB*** **, and** ***pqqL*** **genes.** qPCR was used to determine differential expression under various growth conditions. All transcripts were normalized to *gapA*. (A) Wild-type strain was cultured in LB, LB with dipyridyl and LB with excess iron. In *E. coli* CFT073, *yddA*, *yddB* and *pqqL* genes are not upregulated during iron limitation and are not repressed in the presence of additional iron. (B) Transcript levels in the wild-type and Δ*fur* strain, cultured in LB, were determined using qPCR. Relative quantification of transcripts reveal that lack of Fur results in upregulation of *yddA*, *yddB*, and *pqqL* transcripts, compared to wild-type strain. Mean from three independent qPCR reactions are plotted here. Error bars indicate SEM.
(TIF)

Table S1 **Oligonucleotide primers used in this study.**
(XLSX)

Table S2 **List of candidate bacteremia fitness genes in uropathogenic** ***E. coli*** **.**
(XLSX)
