# Supplementary figures and images for: Genome-Wide Detection of Fitness Genes in Uropathogenic Escherichia coli during Systemic Infection
Source: PLoS Pathog. 2013 Dec 5;9(12):e1003788. doi: 10.1371/journal.ppat.1003788 (PMC3855560; doi:10.1371/journal.ppat.1003788)

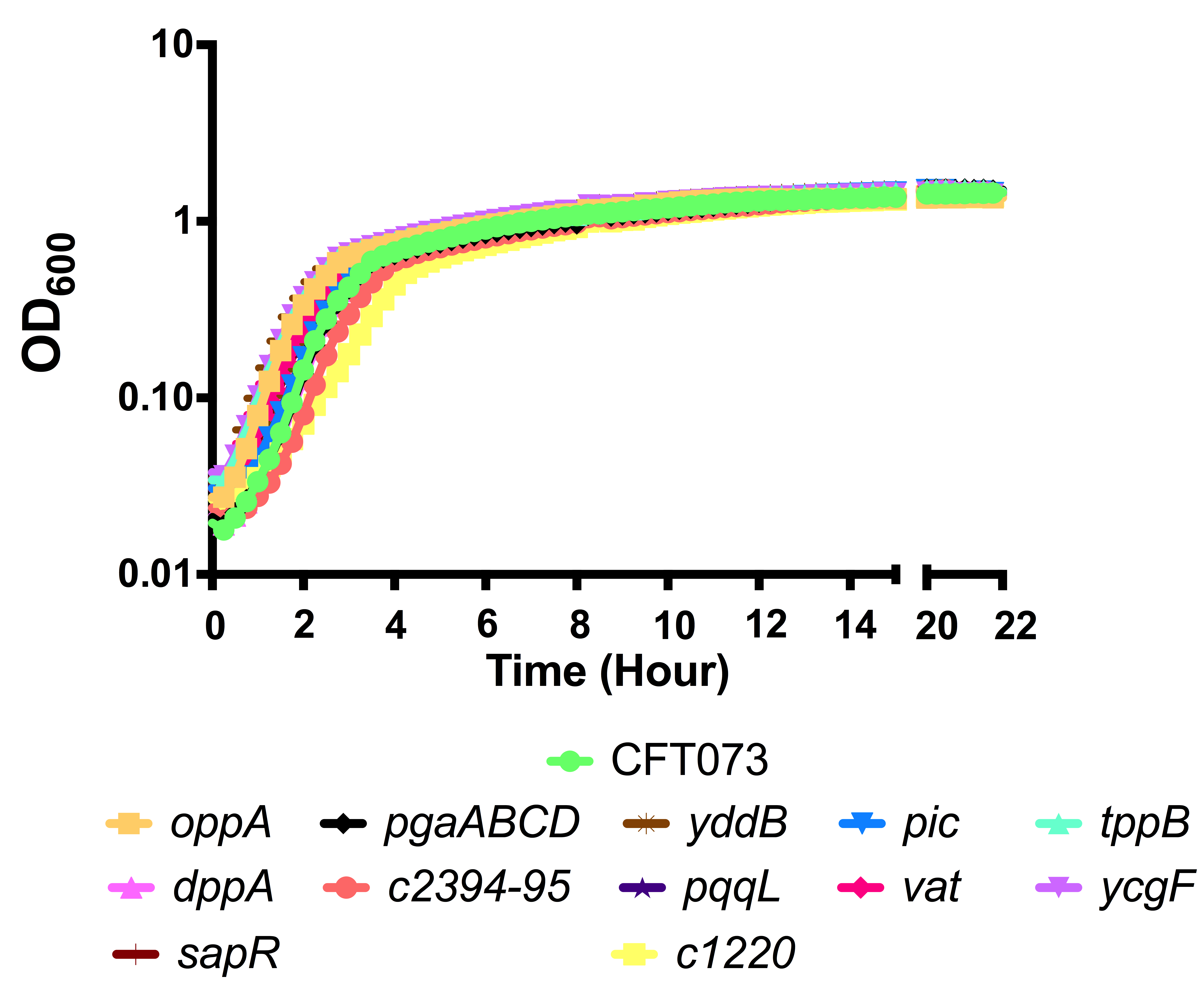

Supplement: Figure S1 — Growth patterns of mutant strains are similar to wild-type strain. Growth kinetics of wild-type and mutant strains, used in co-infection experiments, were determined in LB. Optical density measurements were recorded using a BioscreenC system. Differences in growth pattern are not discernible, indicating that these mutants are not compromised in fitness during growth in LB in vitro. Mean from three independent experiments are plotted here and error bars indicate SEM (not obvious in the figure due to small variation from the mean). (TIFF) [file ppat.1003788.s001.tif]

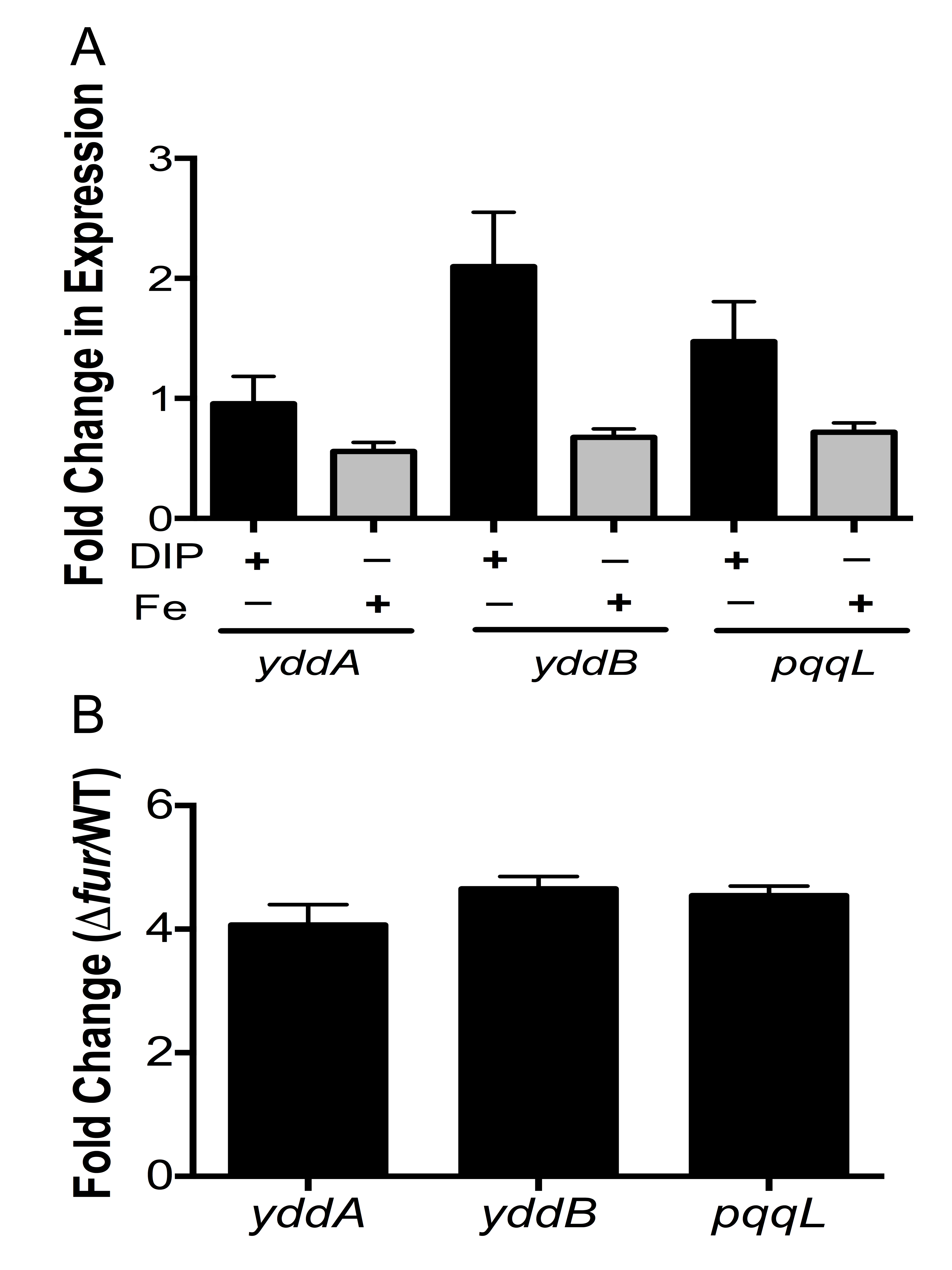

Supplement: Figure S2 — Iron levels do not regulate the expression of yddA , yddB , and pqqL genes. qPCR was used to determine differential expression under various growth conditions. All transcripts were normalized to gapA. (A) Wild-type strain was cultured in LB, LB with dipyridyl and LB with excess iron. In E. coli CFT073, yddA, yddB and pqqL genes are not upregulated during iron limitation and are not repressed in the presence of additional iron. (B) Transcript levels in the wild-type and Δfur strain, cultured in LB, were determined using qPCR. Relative quantification of transcripts reveal that lack of Fur results in upregulation of yddA, yddB, and pqqL transcripts, compared to wild-type strain. Mean from three independent qPCR reactions are plotted here. Error bars indicate SEM. (TIF) [file ppat.1003788.s002.tif]
